# Comparison of Mechanical Properties of Steam-Free and Steam-Based Specimens Made of Expanded Polypropylene Beads

**DOI:** 10.3390/polym16030400

**Published:** 2024-01-31

**Authors:** Sören Handtke, Jörg Hain, Fabian Fischer, Tim Ossowski, Klaus Dröder

**Affiliations:** 1 Volkswagen AG, Berliner Ring 2, 38440 Wolfsburg, Germany; 2 Institute of Machine Tools and Production Technology, Technische Universität Braunschweig, Langer Kamp 19b, 38106 Braunschweig, Germany

**Keywords:** bead foam, expanded polypropylene, steam-free processing technology, material testing

## Abstract

Reducing the CO_2_ emissions of plastic parts is crucial in terms of sustainable product and process designs. Approaches include the use of recycled materials and reducing the energy demands of processes through more efficient technologies. In this context, this study shows the potential of the steam-free processing of particle foam beads into thin-walled moulded parts. Expanded polypropylene (EPP) particle foam beads have been processed in both a steam-free and steam-based process. For this purpose, specimens with different part densities and thicknesses were produced, the mechanical properties were investigated, and the surface quality was discussed. Specimens made of EPP with a part thickness of 5 to 20 mm and part densities of 60 to 185 g/L were produced steam-free. Lower part thicknesses and higher densities increase the mechanical properties. As the density increased, the homogeneity of the surfaces of the steam-free specimens also increased. In comparison, specimens with a thickness of 10 mm and part densities of 35 to 90 g/L were produced on a steam-based process. The results of the mechanical test were compared with those of the steam-free specimens. The steam-based specimens showed higher mechanical properties for the same density.

## 1. Introduction

**Figure 1:**
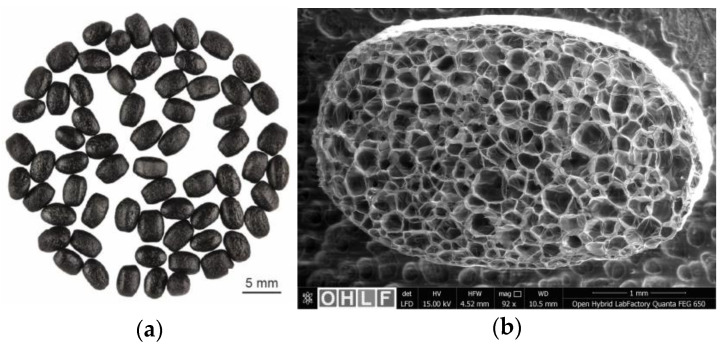
(**a**) Particle foam beads made of polypropylene; (**b**) Inner cell structure of a particle foam bead.

A wide range of industries are currently faced with the challenge of making their products and production more sustainable and transforming them in order to meet sustainability requirements and laws. For example, the European Union’s latest draft legislation calls for the general use of 25% recycled materials for plastic parts in future car models, of which 25% should come from post-consumer recyclate (PCR) [1]. One way to become more sustainable is to reduce the CO_2_ footprint of fossil-based plastic parts. This can be achieved by establishing a material recycling economy or by using plastic parts with low material input. One approach to reducing material consumption in plastic parts is to use lightweight materials in the form of particle foams. These consist of particle foam beads, which have an enormous potential for reduced material use due to their closed microcellular structure and the resulting trapped air content (see Figure 1). With particle foam parts made of expanded polypropylene, moulded part densities in the range of 15–350 g/L are possible [2].

Particle foam parts can play an important role in reducing energy consumption in future vehicle concepts. Alternative drive systems, such as those used in battery-electric vehicles (BEVs), require efficient air conditioning in the passenger cabin in order to maximise the amount of energy available for the drive. For this reason, the thermal insulation effect of particle foam parts due to their closed-cell structure is of interest in order to have a direct positive effect on the thermal balance of a vehicle interior.

## 2. Theories

Polymer foam parts are generally very well suited for compression-absorbing applications, as they can undergo large deformations when absorbing significant specific energy. These properties are also used in automotive applications. In addition, particle foam parts are used in current cars due to their lightweight potential as well as their thermal and acoustic insulation properties [3,4]. Particle foam parts are used, for example, in the exterior area as pedestrian impact protection (bumper cores) and air deflectors, or in the interior area as tool boxes but also as so-called circulating containers (trays) in production and logistics.

But since part volume is a limited resource in automotive applications, it is of interest to make the volume of particle foam parts as low as possible. For this reason, the dependencies of the mechanical characteristics on the sample thickness and density, as well as on the processing method, are discussed. Avalle [4] refers to many experimental studies carried out to determine the compressive properties of steam-based EPP particle foam parts for automotive use [5,6,7,8,9,10,11,12,13,14,15]. It was pointed out that the results are rather inconsistent and not general enough. In general, the compressive properties show a strong dependence on strain rate, but also, in particular, on part density. A higher part density not only shows a higher stress but also a higher energy absorption. For example, a part density of 60 g/L results in three times the stress of a part density of 20 g/L. However, most tests are carried out under quasi-static loading. It was also mentioned that, especially for automotive applications, the behaviour of the foam under dynamic loading with strain rates of up to 100 s^−1^ is of interest.

Expanded particle foam beads are currently processed using a steam-based technology [16]. This allows particle foam parts to be realised with great geometric freedom. The steam-based process is well established in the industry and has been described in numerous publications [16,17,18,19,20,21]. The steam-based process of making particle foam beads is characterised by a hollow chamber tool design and the use of steam for temperature control of the mould and for welding the particle foam beads. The steam pressure curve exhibits an exponential behaviour that corresponds to the temperature-dependent gas pressure of water. A steam pressure of approximately 1.0–1.5 bar is necessary for temperatures ranging from 100 to 115 °C, which is typically used for processing parts containing expanded polystyrene (EPS). For EPP parts, a steam pressure of 3.6 bar is required for temperatures around 140 °C. To process technical thermoplastic beads, such as expanded polybutadiene (EPBT), which has a melting point of around 200 °C, pressures in the upper 20 bar range are necessary. Using water vapour as an energy carrier medium, the temperature required for welding the particle foam beads is transported into the moulded part.

**Figure 2:**
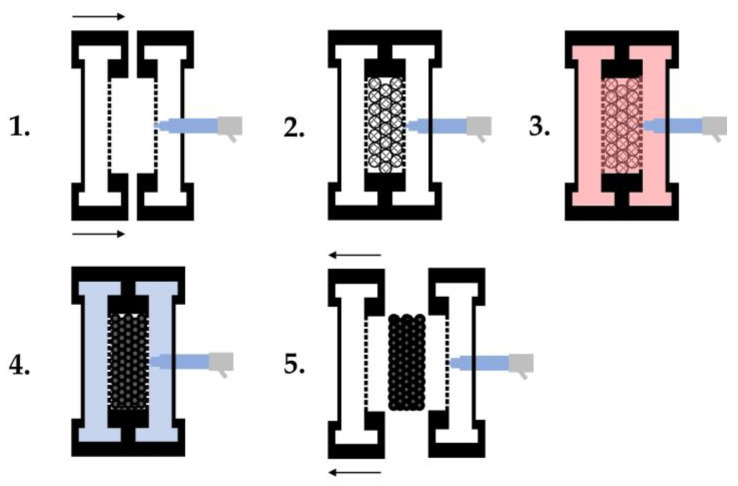
Steam-based processing of particle foam parts based on [16].

The actual process is divided into five steps, as shown in Figure 2. 1. Closing the mould; 2. Filling the cavity with particle foam beads; 3. Steam deposition/welding of the beads; 4. Cooling of the mould; 5. Opening the mould and removing the part. The crucial step in the process of welding the beads consists of three individual steps: 3a. preheating of the mould and venting; 3b. cross-steaming; and 3c. autoclave steaming. In this process, the cavity filled with particle foam beads is compressed in order to create the most direct contact between the beads and each other. The introduction of steam results in the softening of the bead skin and the formation of weak binding forces (van der Waals bonds), which is followed by the formation of the cohesive bead bond. This is achieved by the diffusion of polymer chains across the interface/boundary layer of the neighbouring bead skins [16]. The process of diffusion of polymer chains is necessary to ensure sufficient and uniform welding of the beads over the entire geometry of the moulded part and depends, among other things, on the temperature, the distribution of the chain ends, and the molecular weight, which has been investigated in a large number of studies [22,23,24,25,26,27,28,29].

The processing of the particle foam beads is determined by the temperatures, especially at the interface of each bead, which depend on the thermal properties of the material. Particle foam beads often have a double-melt peak. When processing EPP beads, the steam temperature is set as centrally as possible between these melting peaks. This melts crystalline structures that represent the lower melting range, thus contributing to the sintering of the individual beads. Crystallite structures representing the upper melting range do not melt, thus preserving the cell structure of each bead and the dimensional stability of the foamed part. 

The lower melting range is often referred to as the so-called alpha-1 structure, while the upper melting range consists of the so-called alpha-2 structure. These structures differ in the orientation of the methyl side group from the local axis of the helix structure of polypropylene. In the production of particle foam beads in the autoclave foaming process, the crystal structures mentioned above are achieved by saturation at elevated temperatures in the autoclave (alpha-2 structure) and a subsequent pressure and temperature relaxation (alpha-1 structure) [2,30].

According to Srivastava, the factors influencing the properties of foam parts are, in particular, the polymer type, the ratio of closed to open cell content, the foam density, the foam structure itself, and the use of additives [17]. Reference is made to three publications by Mills et al., which have dealt intensively with the compression behaviour, the Poisson number, and the penetration depth and analysed the relationships between the creep rate, the gas leakage, and the buckling behaviour of the cell structure of polypropylene particle foams [21,31,32]. It should be noted that in the steam-based process, a large number of process parameters influence the physical and mechanical properties of the EPP particle foam parts. Collapse of the beads occurs, especially if the steaming time is too long. As a result, studies of expanded polystyrene (EPS) particle foam parts have shown that the quality of the beads, particularly when welded together (inter/intra-bead bonding), is a decisive failure parameter [33].

**Mechanical characteristics:**

Foam parts are rarely subjected to tensile loads, as they generally exhibit poor behaviour under this type of load and fail easily. Tensile tests on foam specimens are time-consuming, as they have to be clamped in the clamping jaws of the tensile testing machines without destroying the cell structure [21].

The welding quality of specimens (9 × 4 × 15 mm) made of EPP particle foam beads was investigated by Gensel using in-situ tensile tests under a scanning electron microscope (SEM). The tensile strength increased with an increase in the steam pressure and, thus, in the welding temperature. An increase in the steaming duration only led to an increase in the tensile strength values at 3.5 bar steam pressure (corresponding to 140 °C steam temperature), while no significant increase was caused at 2 bar steam pressure (corresponding to 120 °C steam temperature). Following the theory described above, the fracture patterns showed intra-bead fractures only at 140 °C steam temperature, which suggests a cohesive bead bond due to sufficient diffusion of the polymer chains. This assumption was confirmed by the analysis of the fracture surfaces by atomic force microscopy [34].

Morton carried out tensile tests at different temperatures with specimens cut out of automotive parts (30 × 30 × 60 mm^3^ and 30 × 30 × 50 mm^3^) of different densities (28 g/L and 31.3 g/L). Tensile stress-strain curves showed a clear temperature dependence, with Young’s modulus and ultimate stress increasing with lower temperatures. The highest stresses were measured at approximately 0.729 Mpa true stress at −10 °C, while the lowest stresses were measured at <0.1 Mpa at 60 °C [35].

Gebhart carried out tensile tests in accordance with ISO 1926 on EPP specimens with densities of 60 and 80 g/L, milled from plates. Among other things, the degree of crystallinity of the EPP specimens was varied by adjusting the temperature and pressure of the processing process, and the influence on the tensile strength was investigated. With a higher degree of crystallinity, an increase in tensile strength could be demonstrated [36].

Compressive loads are predestined for foams and are a common type of load, e.g., in the packaging industry, due to the weight of the contents [21].

The qualitative stress-deformation curve of a polymer foam sample under compressive loading is divided into three sections. The linear-elastic region up to approx. 5% compression This is followed by the area of plastic deformation, characterised by the transition into a plateau. This area is caused by the onset of the collapse of the beads in the form of bulging, plastic yielding, or brittle behaviour. This curve increases steadily with increasing deformation up to the area of compaction. There, increasing contacts between individual cell walls of the inner cell structure occur [37,38].

Table 1 summarises the publications in which the compression testing of EPP specimens has been performed with various parameters. In some publications, it was not possible to determine all the settings, and this information has been omitted from the table. It can be seen that in each of the publications listed, different specimen/test parameters were used to determine the compression properties of EPP specimens, which makes it difficult to compare the results obtained with each other [4,39]. It can be summarised that with an increase in density, on the one hand, the compaction behaviour was achieved at lower strains, and on the other hand, higher compressive strengths were also measured [40].

According to Mills, a bending load is a common type of load on foam parts. In the 3-point bending test, the tension depends on the geometry, in this case, the thickness of the foam part and thus the distance to the neutral fiber. Failure often occurs on the tensile side of the specimen [21]. There were no characteristics for the three-point-bending properties of EPP specimens found in the literature. Gebhart tested EPP specimens in a four-point-bending test and used the results to compare an FEM Model [36].

As shown above, characteristic values of different specimen dimensions and test procedures for steam-based processed particle foam beads made of polypropylene can be taken from the literature.

The aim of this study is to enable for the first time a direct comparison of the mechanical properties of specimens manufactured with the same EPP material but different processing technologies and tested with the same test settings and procedures. A presentation of the material used (Section 3.1) is followed by a description of the steam-based processing (Section 3.2) and the steam-free processing of EPP specimens (Section 3.3). All specimens were subjected to the same quasi-static mechanical tests (Section 3.4) to determine mechanical properties and to compare the two processing technologies (Section 4.2).

## 3. Materials and Methods

In the following, the material used in this study is briefly presented, and the steam-based and steam-free processing are explained.

### 3.1. Material

**Figure 3:**
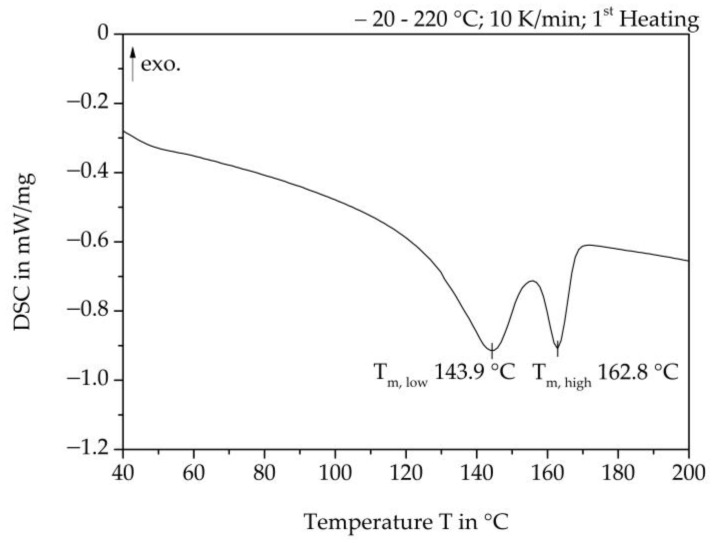
DSC Analysis of EPP Particle foam bead (First heating curve).

For the steam-free and steam-based production of the specimens, particle foam beads made of expanded polypropylene (EPP) with a bulk density of 33–37 g/L were used. EPP particle foam beads usually have two melting peak temperatures. To determine these temperatures, thermal analyses were performed using the method of differential scanning calorimetry (DSC) with the DSC device DSC 204 F1 Phoenix from Netzsch. Figure 3 shows a representative curve of the first heating of the EPP material used. The two melting regions with the melting peak temperatures are clearly visible at T_m,low_ 143.9 °C and T_m,high_ 162.8 °C.

**Figure 4:**
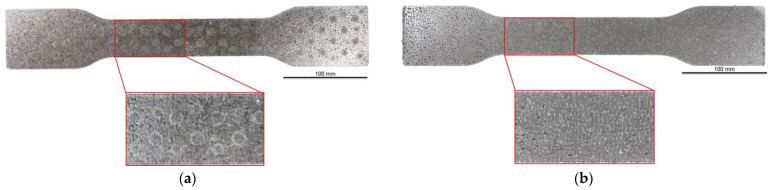
Tension rod made of EPP particle foam beads. (**a**) Steam-based processing, (**b**) Steam-free processing.

As part of this study, EPP particle foam beads were processed into specimens using both steam-free and steam-based processes, and their mechanical properties were tested. Figure 4 shows steam-based and steam-free specimens and close-ups of the surfaces.

### 3.2. Steam-Based Processing

In this study, the steam-based processing of EPP beads was carried out on a steam chest moulding machine to produce specimens with different densities at a part thickness of 10 mm. 

#### 3.2.1. Experimental Design of Steam-Based Processing of EPP

Table 2 shows the experimental design for the production of steam-based specimens. In order to produce specimens with the lowest possible density (between 35 and 40 g/L), pressure loading of the EPP beads is necessary. Depending on the manufacturer’s instructions, the beads are exposed to increased pressure or a pressure curve for a defined period of time. The aim is to compress the beads in the pressure tank and then increase the expansion of the beads during steam-based processing.

#### 3.2.2. Process Parameters

The process of the steam-based processing has already been described in the introduction above. In this study, the steam-based specimens were also produced using the described process of cross-steaming and subsequent autoclave-steaming. Initially, an internal foam pressure of approx. 1.6 bar is achieved through cross-steaming, which drops to less than 1 bar within approx. 10 s. Autoclave steam deposition achieves a maximum steam pressure of approx. 3.5 bar (approx. 140 °C). The pressure is continuously reduced in combination with the cooling of the tool until the end of the process.

### 3.3. Steam-Free Processing

In this study, the steam-free processing of particle foam beads is carried out with a variothermal tool. This is used to produce specimens of different thicknesses and densities for mechanical testing. Since steam is not used as an energy carrier, as in steam-based foaming, heat is transferred by conduction to the centre of the part to soften the bead skins and allow welding to form a bead bond. In this study, no additives or processing aids were used to improve heat conduction from the mould to the core of the part. Possible additives would be those that coat the bead skin, have a higher thermal conductivity than the polymer material and thus improve heat transfer to the core, or soften at a temperature lower than the low melting point of the particle foam bead.

#### 3.3.1. Experimental Design for Steam-Free Processing of EPP

The aim of this study was to demonstrate a dependence of the mechanical characteristics on the thickness and density of steam-free particle foam specimens. The geometry-to-thickness ratio for EPP specimens is shown in Table 3. The minimum and maximum achievable thicknesses and densities, as well as all densities, were produced for a thickness of 10 mm and all thicknesses for a density of 60 and 110 g/L. The production of specimens with a thickness of 15 and 20 mm and a density > 110 g/L was not possible due to the limited height of the cavity.

**Figure 5:**
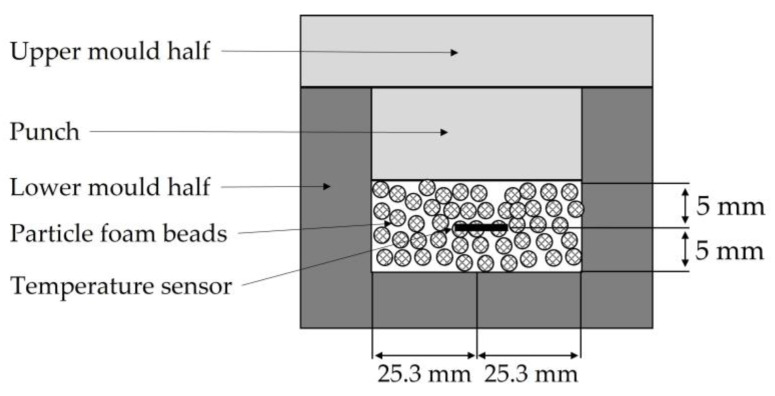
Position of temperature sensor in the moulded part.

In order to quantify the thermal relationships involved in processing expanded particle foam beads in a steam-free, variothermal process, the temperature difference was determined as a function of part thickness. For this purpose, six temperature curves were recorded in the center of gravity of the moulded part using a temperature sensor (PT100); see Figure 5. From these results, predictions can be made for achieving the necessary temperature in the core of the part for a cohesive bead bond. The results are described in Section 4.1.

#### 3.3.2. Tool Design

**Figure 6:**
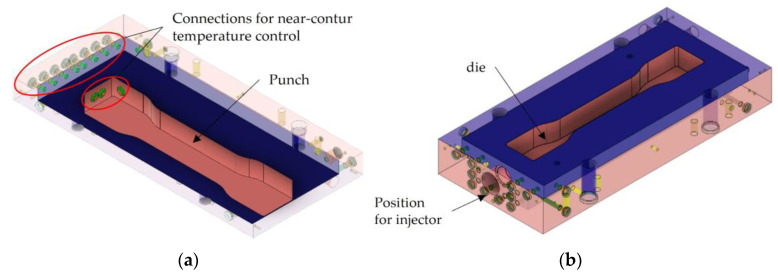
CAD-Concept of the steam-free variothermal tool. (**a**) Upper mould half; and (**b**) lower mould half.

The steam-free variothermal mould is made of steel, is designed as an immersion edge mould and consists of an upper and lower mould half as well as a cavity in the form of a multi-purpose specimen (see Figure 6). Due to the design of the tool with an immersion edge, the processing of already expanded, non-propellant beads is made possible by the gap filling method. Compressed air ejectors for residue-free demoulding of the component and temperature sensors are incorporated into the lower and upper mould halves. The mould is placed in a vertical press that is controlled by software to allow for precise, path-controlled opening and closing of the mould. The temperature control unit consists of two temperature circuits to maintain different temperatures. To ensure that the heating and cooling are as close to the contours as possible and are defined and uniform, the channels are located close to the cavity.

#### 3.3.3. Process Parameters

To produce the steam-free specimens, the mass of EPP beads was weighed and filled into the mould cavity. Following a heating phase, the temperature is maintained for a defined period of time, followed by the cooling phase and the removal of the specimen. The exact parameters are given in Table 4. The temperature used at the start of the holding time for 5 and 10 mm thick specimens is the temperature used in the steam-based process. The holding time in the steam-free process can be compared to that of cross- and autoclave-steaming in the steam-based process. For a part with a thickness of 10 mm and a density of 90 g/L, it takes approximately 30 s.

### 3.4. Mechanical Test Methods

Three mechanical tests are carried out to test the mechanical properties of stem-based and steam-free specimens. Since there is no defined standard for the testing of particle foam specimens, the specimens are tested on the basis of existing standards (see Table 5). All tests were carried out in a standard atmosphere (23 °C). For the bending and compression tests, the specimens were mechanically cut from the center of the manufactured specimens. The specimens were not separately pre-treated in order to test realistic load or application cases.

## 4. Results

First of all, the temperature in the part core during the steam-free variothermal processing of EPP particle foam beads is discussed. The mechanical properties of the steam-free and steam-based specimens, as well as their fractures and surfaces, are discussed in order to evaluate the dependence of the properties on the thickness and density of the specimens. Inter-bead or intra-bead fractures of the EPP specimens can be determined on the basis of the fracture patterns.

### 4.1. Core Temperature in Steam-Free Processing of EPP-Specimens

For the production of particle foam parts, the welding of the individual beads with each other is important. For this purpose, a temperature within the temperature region between the two melting peaks for EPP is sought. As the steam-free process introduces heat into the part by convection and conduction without any additional medium, it is essential that the temperature distribution throughout the part be sufficiently high to ensure good welding of the beads.

The experimental setup for measuring the core temperatures is described in Section 3.3.1. In Table 6, the average core temperatures of the examined mould thicknesses and the temperature difference from the mould wall are listed. It is obvious that using the process settings shown in Table 4, lower core temperatures are achieved as the mould thickness increases. At the same time, the temperature differences from the mould wall to the moulded part core increase as the moulded part thickness increases. It can be seen that only with a moulded part thickness of 5 mm can sufficient temperatures in the region of the first melting peak of the EPP particle foam beads used (Figure 3) be reached. As a result, the outer skins of the beads can melt and form a cohesive bond with each other only at this moulding thickness. Part core temperatures have a direct influence on the failure behaviour of the foamed parts under load, which is demonstrated by mechanical tests.

### 4.2. Mechanical Tests

The results are presented according to the type of mechanical test. For the density variation of the moulded part thickness of 10 mm, the stress-deformation curves and strength characteristics are shown, and then a comparison with the other thicknesses and densities is presented. Subsequently, the mechanical characteristics of the steam-free specimens are compared with those of the steam-based specimens.

#### 4.2.1. Tensile Tests

**Figure 7:**
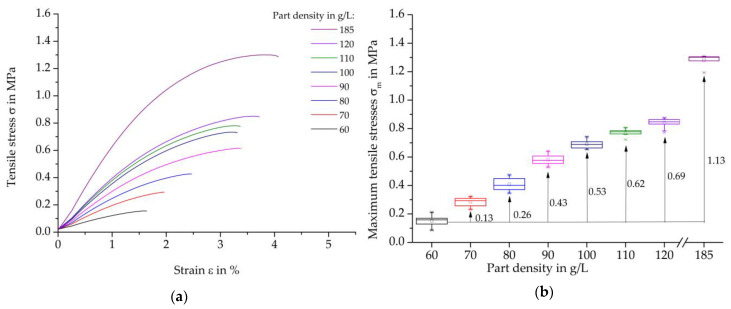
Tensile test results on steam-free EPP specimens with a thickness of 10 mm of varying densities. (**a**) Representative stress-strain curves; (**b**) Maximum strength per density.

Figure 7 shows tensile test results on steam-free EPP specimens with different densities and a thickness of 10 mm. On the left, representative curves of the individual stress-strain curves are shown, and on the right, the maximum strength values as a function of the density and the absolute increases in the average characteristic values in relation to the reference density of 60 g/L of the EPP grade used.

In general, it can be stated that a density of 60 g/L achieves the lowest tensile strength, while the highest density of 185 g/L also has the highest tensile strength values. Every 10 g/L increase in the density of the part leads to a steadily reducing increase in the average maximum strength, resulting in a degressive curve. Furthermore, an increase in the part density by 10 g/L results in a reduction in the standard deviation. This may be due to the fact that the beads are pressed more tightly together as the part density increases, thereby increasing the possibility of better and more homogeneous welding or form-fitting of the beads. Statistically, that can lead to fewer imperfect areas for damage initiation.

**Figure 8:**
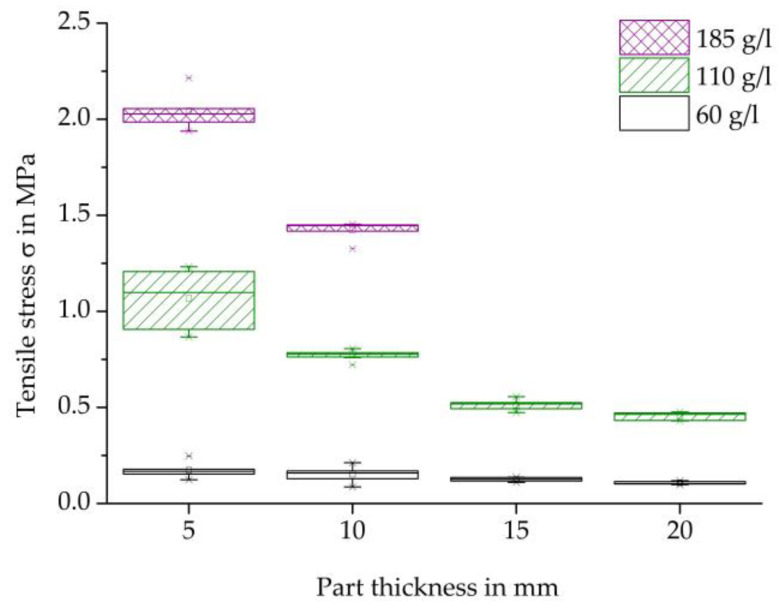
Comparison of tensile strength, of thickness, and density variation of steam-free EPP-Specimens.

Figure 8 clearly demonstrates that as density increases, maximum tensile strength also increases for all tested thicknesses. It is important to note that the increase in characteristic values cannot be solely attributed to force transmission via cohesive connections between the beads due to the lack of complete welding of the particle foam beads in the steam-free specimens. Increasing the density per specimen results in stronger compression of the particle foam beads during processing. This increases the contact area of the bead surfaces, allowing for greater interlocking and the formation of undercuts, which may lead to increased adhesion and force absorption. As the thickness increases, the maximum strength decreases for each specimen density. An increase in thickness per specimen density increases the volume of the specimen, requiring a greater penetration of the temperature profile from the outside into the core of the specimen. The temperature in the core of the test specimen decreases as the thickness of the moulded part and the penetration depth of the temperature increase (see Table 6). This, in turn, means a reduced melting of the bead skins, which prevents a cohesive bead bond. Furthermore, the lower temperature input can lead to less deformation of the beads during the compression step, so that fewer contact surfaces can be formed.

**Figure 9:**
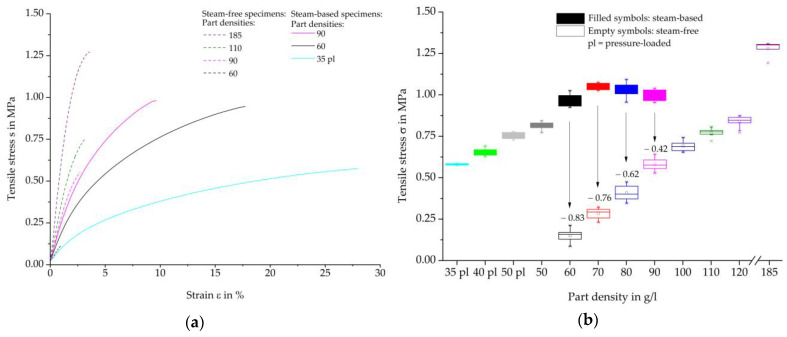
Comparison of tensile characteristics of steam-based and steam-free EPP-specimens with a thickness of 10 mm. (**a**) Representative stress-strain curves; (**b**) Maximum tensile strength per density.

The representative tensile-strain curves of the steam-free (dashed curves) and steam-based (solid curves) specimens are shown in Figure 9a, while Figure 9b shows the resulting tensile strength maxima. The tensile stress-strain curves of the steam-based specimens show a degressive curve based on a linear-elastic increase. Higher density results in steeper gradients, but also lower strain and higher tensile strength maxima. Compared to the steam-based specimens, the steam-free specimens have steeper gradients with lower strain at the same density.

A comparison of the tensile strength maxima of the steam-based specimens shows that there is an initial increase in tensile strength as the density of the moulded parts increases, both in the previously pressure-loaded beads and in the non-pressure-loaded beads. The highest tensile strength is achieved at a density of 70 g/L, which is followed by lower tensile strength maxima as the density increases. The tensile strength of the steam-free specimens described above has lower characteristic values compared to the steam-based specimens. However, as the density increases, the deviations steadily decrease. Thus, the tensile strength of the steam-free specimens at a density of 120 g/L with an average of 0.84 MPa is the same as that of the steam-based specimens at a density of 50 g/L with an average of 0.81 Mpa. However, in our studies, the steam-based process did not allow densities above 90 g/L to be achieved with the selected material type and process.

The steam-based specimens show higher tensile strength maxima and higher elongation at comparable densities, suggesting that the bead bond is more developed than in steam-free specimens.. This allows for a higher and more uniform transmission of force between the beads. The improved bead bond of steam-based specimens may be due to higher core temperatures than those of steam-free specimens. These temperatures can be achieved, regardless of the thickness of the moulded part, by the steam introduced into the cavity filled with beads under high pressure.

#### 4.2.2. Bending Tests

**Figure 10:**
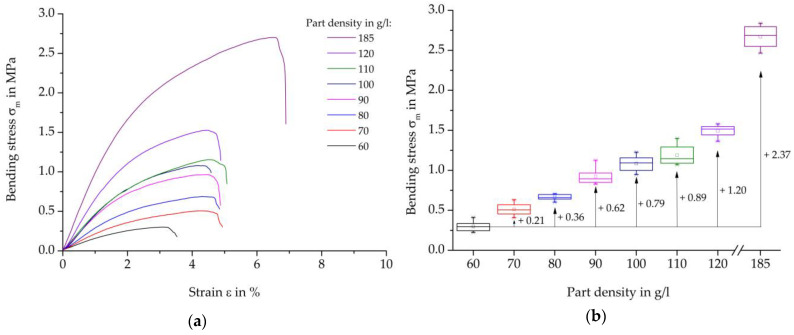
Results of three-Point-Bending test on steam-free EPP specimens with a thickness of 10 mm of varying densities. (**a**) Representative stress-strain curves; (**b**) Maximum strength per density.

Figure 10 shows the results of the three-point bending test on steam-free EPP specimens with a thickness of 10 mm. On the left, representative curves of the individual stress-strain curves are presented, and on the right, the maximum strength values per density are presented in box diagrams. Furthermore, the absolute increases in the average characteristic values in relation to the reference density of 60 g/L are shown.

Analogous to the tensile stress-strain curves, the bending test starts from an almost linear elastic curve and involves the phase of plastic deformation up to the maximum force or failure of the specimen. With an increase in the part density of 10 g/L, the specimens exhibit steadily increasing bending strength.

**Figure 11:**
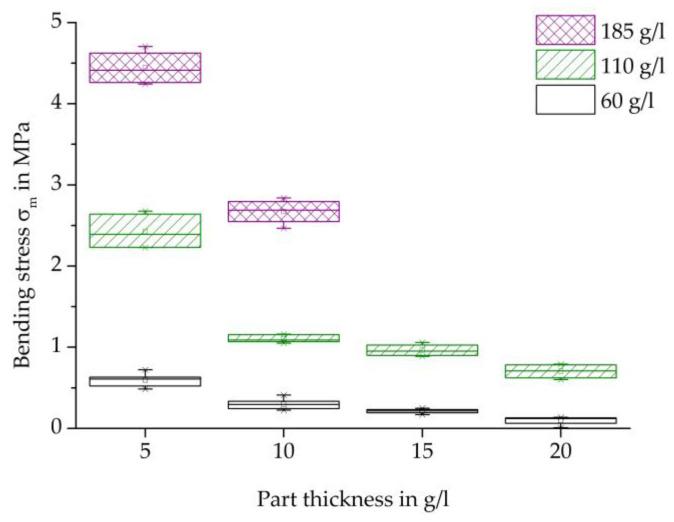
Comparison of the Bending-stess characteristics varying the thickness and density of steam-free EPP-specimens.

Figure 11 shows an overview of the maximum bending strength of each specimen thickness examined and the densities of 60, 110, and 185 g/L.

Analogous to the results of the tensile test, an increase in maximum strength occurs with an increase in density at each specimen thickness. The reasons for the increased strength of the higher densities can also be due to the increased compression and thus more pronounced contact surfaces in connections with partially cohesive areas.

In bending testing, too, the maximum strength decreases with an increase in thickness for each specimen density. This can also be explained by the lower temperature input, so that a weaker bead bond could be created than with a lower thickness.

**Figure 12:**
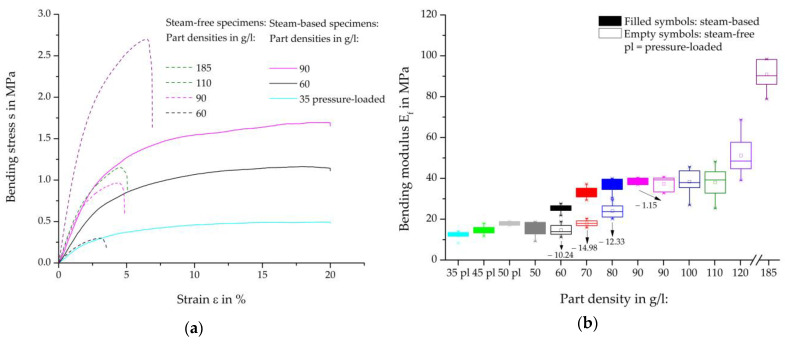
Comparison of bending tets of steam-based and steam-free EPP-specimens with a thickness of 10 mm. (**a**) Representative stress-strain curves; (**b**) bending modulus per density.

Representative bending-strain curves of the steam-free (dashed curves) and steam-based (solid curves) specimens are shown in Figure 12a, and the resulting bending moduli, including the percentage deviation, are shown in Figure 12b. A comparison of the bending maxima of the steam-based specimens shows a divergent result when the maxima are considered. In the curves, force jumps can be seen at 4.5% and at approximately 15% strain, the cause of which can be attributed to the specimen design. Due to the steam nozzle imprints on the specimen surface, these imprints can touch the side of the test punch from a certain deflection, thus resulting in greater force absorption, which is reflected in the force jump mentioned. As a result, the bending moduli were measured at a deformation of 0.35–0.45% (Figure 12b) in order to discuss the comparable characteristics of steam-free and steam-based specimens.

The bending tests of steam-based specimens show strong density-dependent behavior. With increasing density, there is a steeper increase in the linear elastic area and a higher level of plastic deformation, which in total suggests a higher force absorption.

The curves for the steam-free and steam-based specimens show qualitatively significant differences. Noticeable is the much steeper initial increase in steam-free specimens, which implies stiffer behaviour. Furthermore, the failure of the steam-free specimens with lower strain compared to the steam-based specimens is evident. At a density of 185 g/L, the steam-free specimens exhibit a failure with a maximum strain of 6.25%, whereas the steam-based specimens do not fail up to a strain of 20%.

The comparison of the bending moduli of the steam-based specimens (filled symbols) shows a degressive behaviour with an increase in the density of the moulded part. The highest bending moduli are achieved at a moulding density of 90 g/L, which differs from tensile tests. There, the highest parameters were achieved at 70 g/L. The specimens with a moulding density of 50 g/L without prior pressure loading of the particle foam beads show a slightly lower bending modulus than the specimens with the previously pressure-loaded particle foam beads.

Compared to the steam-based specimens, it can be seen that steam-free specimens with the same density achieve lower bending moduli. The bending modulus of the steam-free specimens with a density of 60 g/L is on par with the previously pressurized and steam-based specimens with a density of 35 g/L.

#### 4.2.3. Compression Tests

**Figure 13:**
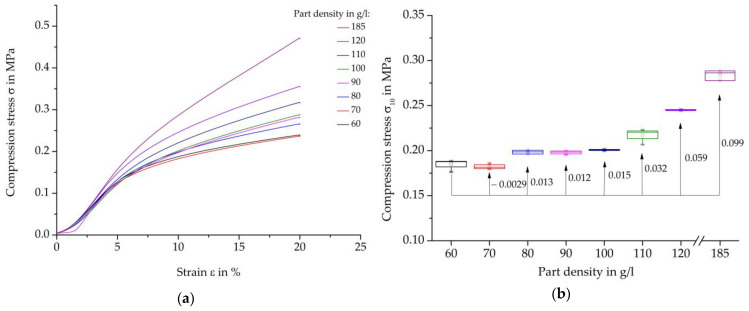
Results of the compression test on steam-free EPP-specimens of different densities. (**a**) Representative stress-strain curves; (**b**) Maximum strength at 10% compression per density.

Figure 13 shows compression test results on steam-free EPP specimens with a thickness of 10 mm. On the left, representative curves of the individual stress-strain curves are presented, and on the right, the strength parameters at a compression of 10% per density are presented in box diagrams. Furthermore, the absolute changes of the average characteristic values in relation to the reference density of 60 g/L are shown.

The curves show the typical behaviour of foam specimens during compression testing, as explained in Section 2. First, there is an approximately linear elastic curve up to about 5% deformation, then the area of plastic deformation, followed by the area of compaction with higher strain. In general, it can also be stated that an increase in density leads to an increase in characteristic values or energy absorption. However, at a compression rate of 10%, this behaviour is not yet strongly pronounced at all densities and only develops with increasing deformation.

Overall, compression strength values do not follow a linear progression. However, a significant trend in terms of increasing strength values with increasing part density can be observed. Up to a part density of 100 g/L, an increase of 8.46% has been achieved in relation to the lowest part density of 60 g/L. The slight reduction of the characteristic values by 1.58% is noticeable at a part density of 70 g/L. The increase in mechanical characteristic values from a part density of 120 g/L to 185 g/L is also small at 15.98% compared to the other test methods.

**Figure 14:**
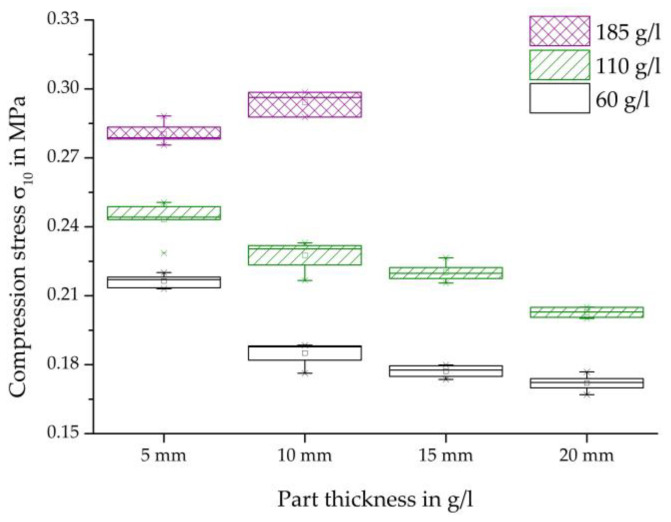
Comparison of the compression strength, varying thickness, and density of steam-free EPP-specimens.

Figure 14 shows an overview of the compressive strength at a deformation of 10% of each specimen thickness examined and densities of 60, 110, and 185 g/L.

In general, these results can also confirm the dependencies of the characteristic values on the density and thickness of the moulded parts shown in the comparisons of other mechanical tests shown above.

As the density increases at each specimen thickness, the maximum strength increases. Similarly, with an increase in thickness, there is a decrease in the maximum compressive strength for each specimen density, with the exception of the increase in thickness from 5 mm to 10 mm. The increased characteristic values at a density of 185 g/L and a thickness of 10 mm compared to the thickness of 5 mm do not fit into the general course or trend of the characteristic values for the other densities and other test methods examined.

**Figure 15:**
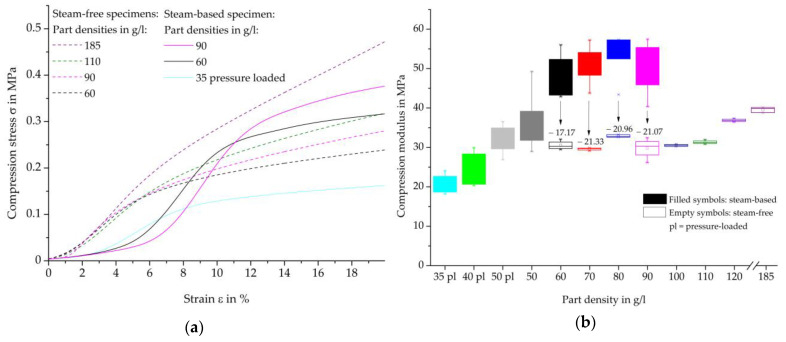
Comparison of compression testing of steam-based and steam-free EPP-specimens with a thickness of 10 mm. (**a**) Stess-deformation curves; (**b**) Compression moduli per density.

Representative compression-deformation curves of individual densities of the steam-free (dashed curves) and steam-based (solid curves) specimens are shown in Figure 15a, and in Figure 15b, the resulting compression moduli. According to the standard, the maximum compression should be determined at a compression of 10%. However, in order to compare the compression maxima of steam-free and steam-based specimens, the value at a deformation of 10% must be reviewed critically. The steam-free specimens are already in the region of plastic deformation, while some densities of the steam-based specimens are at the transition from the linear-elastic to the plastic region. For this reason, and in order to provide the best possible comparability of the results, the compression moduli for the steam-free specimens at a deformation of 3–4% and for the steam-based specimens at a deformation of 5.5–6.5% (densities of 35, 40, and 50 g/L) and 8–9% (densities of 60, 70, 80, and 90 g/L) are shown in Figure 15b.

The compression test of the steam-based specimens shows strong density-dependent behaviour. With increasing density, a higher level of plastic deformation is achieved, which in total suggests a higher force absorption. The curves of the steam-based samples can be divided into two groups. The curves of the moulding part densities of 35, 40, and 50 g/L show an earlier onset of the linear-elastic region, which changes into the region of plastic deformation at a lower strain than the specimens of the moulding densities of 60, 70, 80, and 90 g/L.

The stress-strain curves of the steam-free and steam-based specimens show a high level of qualitative similarity. In the case of the steam-free specimens, the areas of linear-elastic behaviour and plastic deformation occur at lower deformations than those of the steam-based specimens.

The comparison of the compression moduli of the steam-based specimens (filled symbols) shows a degressive and, after reaching the highest bending moduli, a decreasing behaviour at a moulding part density of 80 g/L. The different behaviour of the specimens with moulding part densities up to 50 g/L and 60 g/L is also recognisable here. In contrast to the bending moduli, the specimens with a moulding density of 50 g/L without prior pressure loading of the particle foam beads have slightly higher characteristic values than the specimens of the previously pressure-loaded particle foam beads.

The steam-free specimens (unfilled symbols) achieve lower compression moduli than the steam-based specimens at the same part densities. A significant increase in the number of compression moduli only occurs at a part density of 120 g/L. The compression moduli of the steam-free specimens with a density of 60 g/L are almost at the same level as the moduli of the steam-based and previously pressure-loaded specimens with a density of 50 g/L.

In summary, steam-based specimens achieve higher mechanical characteristics compared to steam-free specimens of the same moulding density. The most decisive parameter in the loading of particle foam moulding parts is the force transmission of particle foam beads to each other. This requires a cohesive bead bond, which can only form when the bead skins fuse. This assumes mould core temperatures above the melting temperatures of the bead skin. As can be seen in Table 6, these temperatures could not be achieved over the entire thickness of the part during steam-free processing, resulting in a lack of cohesive or adhesive bead bonding and consequently lower mechanical characteristics.

### 4.3. Optical Analysis

**Figure 16:**
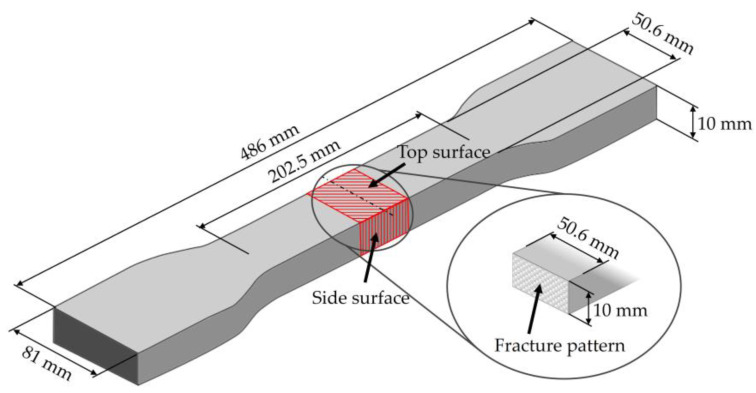
Specimen and locations for the sections for optical analysis.

The top surfaces, side surfaces, and fracture patterns of steam-free EPP specimens of selected densities are comparatively shown in Table 7. The positions of the sections in the specimen are shown in Figure 16.

For example, with a thickness of 10 mm and increasing density, the surfaces of the specimens produced show a higher compression and, at the same time, a smaller number of gaps. At a density of 185 g/L, gaps are only occasionally visible. Due to the high compression of the beads at this density, the beads have a larger contact with the tempered mould wall, which facilitates the formation of a cohesive bond in the outer layer of the specimen. In the case of thicknesses of 5, 15, and 20 mm, a qualitatively similar behaviour occurs with increasing density.

The side surfaces of the steam-free EPP specimens have strongly pronounced gaps, analogous to the images of the low-density surface. These decrease with increasing density. From a density of 110 g/L, it can be seen that the upper layers of EPP beads are more compressed than the lower layers.

In the case of the fracture surfaces of the steam-free specimens, a stronger compression of the individual particle foam beads can be seen with increasing density. At a density of 110 g/L, isolated intra-bead fractures can be seen, indicating that a cohesive bond must have formed with the neighbouring particle foam beads. This is particularly evident in the specimens with a low thickness of 5 mm. These fracture patterns confirm the maximum temperatures in the specimen core shown in Section 4.1. At a maximum temperature of 140 °C with a specimen thickness of 5 mm, the crystallite regions of the alpha-1 structure that correspond to the lower melting area can soften so that a bond with neighbouring beads can be formed.

## 5. Conclusions

In this study, a comparison of the mechanical properties of steam-free and steam-based specimens was carried out on the basis of the same dimensions and test conditions. The steam-free processing of expanded polypropylene particle foam beads into specimens was investigated by varying the thickness and density of the foamed part. Part thicknesses from 5 to 20 mm and part densities from 60 to 185 g/L could be produced. The mechanical characteristics for tensile, bending, and compressive loads showed strong dependencies on the thickness and density of the foamed part.

Low part thicknesses as well as high part densities led to an increase in the mechanical properties. For example, with a part density of 110 g/L, a reduction in the part thickness from 10 mm to 5 mm resulted in an increase in bending strength of 1.3 MPa to 2.4 MPa. An increase in the part density from 60 g/L to 110 g/L, in contrast, resulted in an increase in the bending strength from 0.8 MPa to 1.1 MPa for a part thickness of 10 mm.

The comparison of steam-based specimens with steam-free specimens shows two differences worth mentioning. On the one hand, steam-based specimens achieve higher mechanical properties than steam-free specimens with the same part density. With a part thickness of 10 mm and a part density of 70 g/L, the bending modulus of steam-based specimens is 45% higher than the bending modulus of steam-free specimens. On the other hand, the mechanical properties of steam-free specimens increase with an increase in the density of the foamed parts in all mechanical tests performed, while the mechanical properties of the steam-based specimens decrease again with high part densities under tensile and compressive loads. For further studies, the tool will be modified to produce specimens with higher part densities at thicknesses of 15 mm and 20 mm.

Whether a steam-free processing method for particle foam beads meets the requirements for a more sustainable process technology compared to the steam-based method (e.g., reduction of energy requirements) needs to be determined in further studies.

## Figures and Tables

**Table 1:** Overview Literature Compression test on steam-based EPP specimens.

Author	Standard	Specimen Dimensions [mm]	Densities [g/L]	Preforce [N]	Test Velocity	Number Specimens
Morton [35]	/	30 × 30 × 30; 25 × 25 × 25	28; 31.3	/	/	3
Himmelsbach [40]	/	40 × 40 × 20	30; 60; 120; 210	5	10% H_0_/min	/
Zhang [7]	ASTM D 1621	50 × 50 × 50	/	/	/	/
Avalle [4]	ASTM D 1621	50 × 50 × 50	31; 45; 70; 106; 145;	/	60 mm/min	/
Andena [39]	/	13 × 13 × 13	20; 35; 55; 60; 75; 90; 110; 120	/	/	5

**Table 2:** Experimental design of steam-based EPP-specimens with a thickness of 10 mm.

Density [g/L]	35	40	50	60	70	80	90
Pressure Loading	yes	yes	yes|no	no	no	no	no

**Table 3:** Experimental design of steam-free EPP specimens.

		Part Density [g/L]							
		60	70	80	90	100	110	120	185
Thickness [mm]	5	x					x		
	10	x	x	x	x	x	x	x	x
	15	x					x		
	20	x					x		

**Table 4:** Settings of steam-free process.

Parameters	Settings			
Thickness of specimens [mm]	5	10	15	20
Filling temperature [°C]	75.0	75.0	75.0	75.0
Temperature Start Holding Time [°C]	140.0	140.0	135.0	130.0
Temperature End Holding Time [°C]	142.5	142.5	142.5	142.5
Demoulding Temperature [°C]	80.0	80.0	80.0	80.0
Holding time [s]	60	60	120	180

**Table 5:** Settings used in quasi-static test methods.

Property	Tensile Properties	Compression Properties	Bending Properties (3PB)
Standard (based on)	DIN EN ISO 527-1	ISO 844	ISO 1209
Specimen dimensions	Tension rod	50 × 50 × (5, 10, 15, 20) mm	120 × 25 × (5, 10, 15, 20) mm
Pre-force	5 N	10 N	1 N
Inspection speed	50 mm/min	10% of thickness in mm/min	10 mm/min
End of test	Force Drop > 40%	Compression 20%	Elongation 20%
Characteristic value	Maximum force	Compressive strength at 10% compression, compression modulus	Maximum force, Bending modulus
Number of specimens	8	4	8

**Table 6:** Average of maximum core temperatures of steam-free EPP specimens.

Thickness [mm]	Averaged Maximum Core Temperature of Specimens [°C]		Temperature Difference Core <-> Tool [°C]	
	Absolute Temperature [°C]	Standard Deviation of Core Temperature [°C]	Absolute in °C	Standard Deviation of Core Temperature [°C]
5	139.85	0.5	3.07	0.45
10	129.38	1.26	14.5	0.77
20	119.42	1.96	25.83	0.38

**Table 7:** Surfaces, side surfaces, and fracture patterns of steam-free EPP specimens of selected densities.

		Part Density in g/L		
Type of Area	Part Thickness in mm	60	110	185
Top surface	5	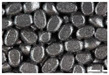	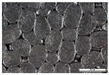	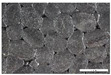
	10	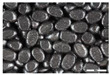	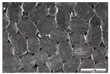	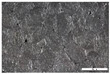
	20	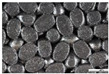	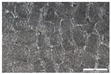	/
Side surface	5	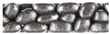	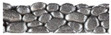	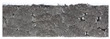
	10	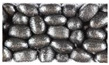	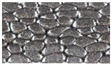	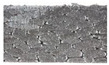
	20	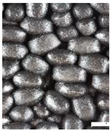	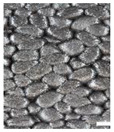	/
Fracture surface	5	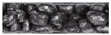	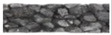	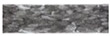
	10	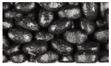	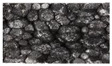	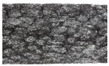
	20	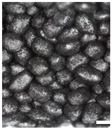	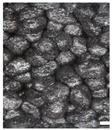	/

## Data Availability

The raw/processed data required to reproduce these findings cannot be shared at this time, as the data also form part of an ongoing study.
